# Specific Temporal Distribution and Subcellular Localization of a Functional Vesicular Nucleotide Transporter (VNUT) in Cerebellar Granule Neurons

**DOI:** 10.3389/fphar.2017.00951

**Published:** 2017-12-22

**Authors:** Aida Menéndez-Méndez, Juan I. Díaz-Hernández, Felipe Ortega, Javier Gualix, Rosa Gómez-Villafuertes, María T. Miras-Portugal

**Affiliations:** ^1^Department of Biochemistry and Molecular Biology, Faculty of Veterinary, Complutense University of Madrid, Madrid, Spain; ^2^University Institute of Neurochemistry Research (IUIN), Complutense University of Madrid, Madrid, Spain; ^3^Instituto de Investigación Sanitaria del Hospital Clínico San Carlos, Madrid, Spain

**Keywords:** VNUT, granule cells, cerebellum, ATP exocytosis, VGLUT1, GCPs

## Abstract

Adenosine triphosphate (ATP) is an important extracellular neurotransmitter that participates in several critical processes like cell differentiation, neuroprotection or axon guidance. Prior to its exocytosis, ATP must be stored in secretory vesicles, a process that is mediated by the Vesicular Nucleotide Transporter (VNUT). This transporter has been identified as the product of the SLC17A9 gene and it is prominently expressed in discrete brain areas, including the cerebellum. The main population of cerebellar neurons, the glutamatergic granule neurons, depends on purinergic signaling to trigger neuroprotective responses. However, while nucleotide receptors like P2X7 and P2Y13 are known to be involved in neuroprotection, the mechanisms that regulate ATP release in relation to such events are less clearly understood. In this work, we demonstrate that cerebellar granule cells express a functional VNUT that is involved in the regulation of ATP exocytosis. Numerous vesicles loaded with this nucleotide can be detected in these granule cells and are staining by the fluorescent ATP-marker, quinacrine. High potassium stimulation reduces quinacrine fluorescence in granule cells, indicating they release ATP via calcium dependent exocytosis. Specific subcellular markers were used to assess the localization of VNUT in granule cells, and the transporter was detected in both the axonal and somatodendritic compartments, most predominantly in the latter. However, co-localization with the specific lysosomal marker LAMP-1 indicated that VNUT can also be found in non-synaptic vesicles, such as lysosomes. Interestingly, the weak co-localization between VNUT and VGLUT1 suggests that the ATP and glutamate vesicle pools are segregated, as also observed in the cerebellar cortex. During post-natal cerebellar development, VNUT is found in granule cell precursors, co-localizing with markers of immature cells like doublecortin, suggesting that this transporter may be implicated in the initial stages of granule cell development.

## Introduction

Extracellular adenosine triphosphate (ATP) and nucleotide analogs influence essential activities in the nervous system, acting through both G protein-coupled P2Y receptors and ionotropic P2X receptors (for review see (Burnstock, 2006, 2013). ATP is mainly released from neural cells by an exocytotic process (Gutierrez-Martin et al., 2011), which requires a previous storage into secretory vesicles (SVs), a process mediated by a specific vesicular transporter. Depending on the cell type and the environmental conditions, ATP release may also be driven by connexins and pannexins, large-pore ion and metabolite channels (for review see (Baroja-Mazo et al., 2013).

Vesicular neurotransmitter transporters (VNTs) drive the storage of specific soluble compounds in SVs, a crucial step in neurotransmission. Vesicles containing more than one neurotransmitter require specific VNTs for each of them. The abundance of ATP in secretory granules opens new horizons in neurotransmission and the term co-transmission was coined by Burnstock (1976, 2004) 40 years ago in reference to such complexity. Initially, the storage of different nucleotides in the same catecholaminergic chromaffin granules was reported, and then further confirmed in cholinergic and other storage vesicles (reviewed by Winkler and Westhead, 1980; Volknandt and Zimmermann, 1986). The vesicular monoamine transporter (VMAT1) was the first VNT to be isolated from a rat leukemia cell line cDNA library (Erickson et al., 1992). The existence of an active vesicular nucleotide transporter (VNUT) was first inferred from its kinetic parameters, exhibiting a mnemonic saturation profile and low specificity, capable of transporting a variety of nucleotides at intravesicular concentrations close to 150 mM (Bankston and Guidotti, 1996; Gualix et al., 1996, 1999a). The VNUT was identified as a product of the *SLC17A9* gene (Sawada et al., 2008), a member of the anion solute carrier transporters 17 family (SLC17) that includes other VNTs, such as the three vesicular glutamate transporter isoforms (VGLUT1, VGLUT2 and VGLUT3). Recently, the role of VNUT has been studied extensively in chromaffin granules from the adrenal medulla, assessing catecholamine storage and granule release (Estevez-Herrera et al., 2016). Moreover, co-localization of VNUT and the vesicular acetylcholine transporter (VAChT) has been reported in cholinergic vesicles from the electroplaque of *Torpedo californica* (Li and Harlow, 2014), and in other types of secretory granules (Haanes and Novak, 2010).

Immunohistochemical studies in the brain have demonstrated that VNUT is not homogeneously expressed in all cell subtypes and anatomical structures, rather it is particularly abundant in the mouse cerebellar cortex (Larsson et al., 2012). Among the different cell populations that constitute the cerebellum, granule neurons in the granular layer of adults is the most numerous cell subtype. Cerebellar granule neurons are glutamatergic cells that express high levels of VGLUT1 (Hallberg et al., 2006). The axons from these neurons ascend to the molecular layer, where they bifurcate to form the parallel fibers that connect with the dendritic tree of Purkinje cells (Ramón y Cajal, 1889, 1904, 1995; Cerminara et al., 2015). Primary cultures of cerebellar granule neurons are widely used to study neurotransmission, as these cells can develop the morphological, biochemical and electrophysiological characteristics of mature neurons *in vitro*. Indeed, they express a variety of functional P2Y and P2X nucleotide receptors, making them a suitable model to study the purinergic system (Hervas et al., 2003, 2005; Leon et al., 2006; Sanchez-Nogueiro et al., 2009, 2014).

The maturation of granule cells in culture is accompanied by time-dependent changes in the expression and activity of purinergic receptors (Sanz et al., 1996; Hervas et al., 2003). We previously reported that activation of either P2X3 or P2X7 receptors induces an increase in cytosolic Ca^2+^ in cerebellar granule neurons, resulting in the exocytotic release of glutamate and the subsequent enhancement of synapsin-1 phosphorylation (Leon et al., 2006, 2008). In addition, both the P2Y_13_ and P2X7 receptors in granule neurons are involved in neuroprotection (Ortega et al., 2009; Ortega et al., 2010, 2011; Morente et al., 2014; Miras-Portugal et al., 2016). Despite the obvious importance of the purinergic system in the pathophysiological context of granule cells, there is currently no information concerning vesicular ATP storage and its relationship with VNUT expression. Moreover, granule neuron development occurs in the context of the cerebellar structure, and thus, it would be relevant to study the presence and evolution of VNUT in a more physiological context.

In the present work, we analyzed the vesicular release of ATP from primary cultures of cerebellar granule cells, studying the involvement of VNUT in this process and the effect of its inhibition. The distribution of VNUT was compared to that of VGLUT1 *in vitro* and *in vivo*, assessing both the subcellular location and temporal evolution during neuronal maturation. Our findings shed light on the ATPergic and glutamatergic nature of cerebellar granule neurons.

## Materials and Methods

### Ethics Statement

All animal procedures were carried out at the Complutense University of Madrid in accordance with European and Spanish regulations (2010/63/EU; RD1201/2005; RD 53/2013), and following the guidelines of the International Council for the Laboratory Animal Science. The C57BL/6 mice used in these studies were obtained in-house. The protocols employed were approved by the Committee for Animal Experiments at the Complutense University of Madrid and by the Committee for Animal Ethics of the Regional Government of Madrid.

### Cerebellar Granule Cells Culture

Primary cultures of cerebellar granule cells were prepared using a modified version of the procedure described by Pons et al. (2001). Briefly, the cerebellum was dissected out from postnatal day 5 mice and dissociated with the Papain Dissociation System (Worthington Biochemical, Lakewood, NJ, United States). Neurons were plated onto coverslips at a density of 120,000 cells/cm^2^ or in culture plates pre-coated with poly-L-lysine (10 μg/ml; Biochrom, Berlin, Germany) and they were maintained at 37°C in a humidified atmosphere containing 5% CO_2_ and in Neurobasal medium (Life Technologies, Gaithersburg, MD, United States) supplemented with 2% B27, 25 mM KCl, 1% GlutaMAX^TM^, 100 U/ml penicillin and 0.1 mg/ml streptomycin. The antimitotic drug cytosine arabinoside (AraC, 10 μM) was added to the culture medium to avoid the proliferation of non-neuronal cells.

### RT-PCR and Quantitative Real-Time PCR

Total RNA was obtained from cultured granule cells using a SpeedTools Total RNA Extraction kit (Biotools), following the manufacturer’s instructions. After purification, the total RNA was quantified and 1 μg was reversed transcribed using M-MLV reverse transcriptase, 6 μg of random primers and 350 μM dNTPs (all from Thermo Fisher Scientific). Quantitative real-time PCR (qPCR) reactions were performed in final volume of 25 μl using the LuminoCt qPCR Ready Mix (Sigma–Aldrich), 5 μl of the cDNA synthesized previously and 1.25 μl of specific commercial TaqMan^®^ gene expression assays for mouse: VGLUT1 (Mm00812886_m1), VNUT (Mm00805914_m1), the housekeeping gene glyceraldehyde 3-phospate dehydrogenase (GAPDH, Mm99999915_g1), all from Applied Biosystems. The qPCR assays were carried out using a StepOnePlus Real-Time PCR System (Applied Biosystems) as follows: denaturation at 95°C for 20 s, followed by 40 cycles of 95°C for 1 s and 60°C for 20 s. As indicated, the expression of each gene was normalized to that of the endogenous housekeeping gene GAPDH amplified in parallel.

### Western Blotting

Granule cells or cerebellar tissues were lysed and homogenized for 1 h at 4°C in lysis buffer (pH 7.4) containing 50 mM Tris-HCl, 150 mM NaCl, 1% Nonidet P40 and Complete^TM^ Protease Inhibitor Cocktail Tablets (Roche Diagnostics GmbH). The cell lysate was then centrifuged at 16,000 × g for 9 min and the supernatants collected. Protein extracts were resolved on 10% SDS-PAGE gels and transferred to nitrocellulose membranes, which were then blocked for 1 h at RT (∼25°C) with 3% BSA in TBS (10 mM Tris, 137 mM NaCl, 2.7 mM KCl, 5 mM Na_2_HPO_4_, 1.4 mM KH_2_PO_4_ and 0.1% Tween [pH 7.5]). The membranes were probed with primary antibodies raised against VNUT (1:500, MBL), anti-VGLUT1 (1:7500, Synaptic Systems) and GAPDH (1:10,000, Sigma Aldrich). Antibody binding was detected by ECL chemiluminescence (Amersham GE Healthcare), and images were captured with ImageQuant LAS 500 (GE Healthcare Life Sciences) and analyzed using ImageQuant TL.

### Immunofluorescence

Granule cells cultured on coverslips in 35 mm dishes were fixed for 15 min with 4% paraformaldehyde (PFA) and then rinsed with PBS twice for 10 min. For immunofluorescence studies of the cerebellum, the extracted brains were fixed in 4% PFA for 24 h, cryoprotected in 30% sucrose, embedded in O.C.T. (Sakura Finetek), and cryosections (30 μM) were obtained in the sagittal plane on a Leica CM1950 cryostat. Afterward, coverslips or cryosections were blocked and permeabilized for 1h at RT with blocking solution (0.1% Triton X-100, 5% FBS and 10% BSA in PBS). The coverslips or sections were then incubated overnight at 4°C with the corresponding primary antibodies: rabbit anti-VNUT (1:100, MBL), mouse anti-βIII-tubulin (1:1000, Promega), mouse anti-VGLUT1 (1:500, Synaptic Systems), mouse anti-synaptophysin (1:500, Sigma–Aldrich), chicken anti-MAP2 (1:1000, Abcam), guinea pig anti-DCX (1:500, Millipore), mouse anti-Math1 (1:250, Abcam), mouse anti-LAMP-1 (1:50, ThermoFisher Scientific), and mouse anti-SMI 312 (1:500, Abcam). The following day, the cells or sections were washed three times with PBS and incubated with the corresponding fluorescent-tagged secondary antibodies for 1 h at RT: Alexa fluor 594^®^ donkey anti-rabbit, Alexa fluor 488^®^ goat anti-mouse, Alexa fluor 647^®^ goat anti-mouse (all from Thermo Fisher Scientific), Cy^TM^5 donkey anti-guinea pig, and FITC donkey anti-chicken (both from Jackson ImmunoResearch). The nuclei were counterstained with 4′, 6-diamidino-2-phenylindole (DAPI, Thermo Fisher Scientific) and the coverslips or sections were finally mounted on glass slides using FluoroSave^TM^ Reagent (Calbiochem). When antibodies against VNUT and Math1 were used on cerebellar sections, streptavidin-biotin amplification of the fluorescence signal was used. Images were acquired on a Leica TCS SPE confocal microscope using the 40X and 63X W/IR objectives.

### Fluorescent Labeling of Vesicular ATP

Quinacrine [4-*N*-(6-chloro-2-methoxyacridin-9-yl)-1-*N,1-N*-diethylpentane-1,4-diamine] was used to visualize vesicular ATP, a fluorescent dye widely used to detect ATP-enriched vesicles (Gutierrez-Martin et al., 2011). Quinacrine staining was carried out by incubating granule cells for 15 min at 37°C in Locke’s buffer (140 mM NaCl, 4.5 mM KCl, 2.5 mM CaCl_2_, 1.2 mM KH_2_PO_4_, 1.2 mM MgSO_4_, 5.5 mM glucose and 10 mM HEPES [pH 7.4]) containing 4 μM quinacrine (Sigma–Aldrich). Time-lapse studies were performed with an Olympus IX81 inverted microscope equipped with a 100X, 1.45 NA, oil-immersion objective. Images were acquired every 200 ms with a Hamamatsu C9100 EM-CCD digital camera (Hamamatsu) controlled by CellR software (Olympus). Vesicular fusion and release into the extracellular space was observed as a rapid loss of the quinacrine signal. During the assays, cells were continuously perfused with Locke’s buffer at a rate of 1.5 ml/min at 37°C. Perfusion was gravity-driven and solution exchange was performed by manually operating electronic valves of a VC-6 drug application system (Warner Instruments). Images were processed using the ImageJ free software v.1.50i (National Institutes of Health, Bethesda, MD, United States).

### ATP Release

Adenosine triphosphate release was measured using the ENLITEN^®^ rLuciferase/Luciferin reagent (Promega). Granule cells were maintained for 30 min at 37°C in Mg^2+^-free Locke’s buffer containing 100 μM ARL67156, a competitive inhibitor of ecto-ATPases (Levesque et al., 2007), and subsequently, 50 μl of extracellular medium was collected to measure the basal ATP levels. The cells were then stimulated with 10 μM ionomycin and after 5 min, 50 μl of the extracellular medium was collected to measure ATP concentration after stimulation. The samples were centrifuged at 600 × *g* for 5 min at 4°C and 10 μl of supernatant were transferred onto 96-well plate placed on ice. When indicated, cells were incubated with 2 μM Evans Blue (Sigma–Aldrich) for 1 h (Loiola and Ventura, 2011). The plate was then situated in a FLUOstar OPTIMA Microplate Luminometer (BMG LABTECH GmbH) and 100 μl of rLuciferase/Luciferin reagent was automatically injected into each well at RT. The ATP concentration was estimated by interpolation from a linear standard curve.

### Statistical Analysis

The data shown are mean values ± standard error of the mean (s.e.m) and all independent experiments shown were reproduced 3–6 times. The figures were generated and the statistical analyses performed using GraphPad Prism 6 (GraphPad Software). The results were analyzed using an unpaired Student’s *t*-tests or ANOVA with Dunnet’s Multiple Comparisons test. A *p*-value ≤ 0.05 was considered statistically significant.

## Results

### Cerebellar Granule Cells Express Native Functional VNUT

**FIGURE 1:**
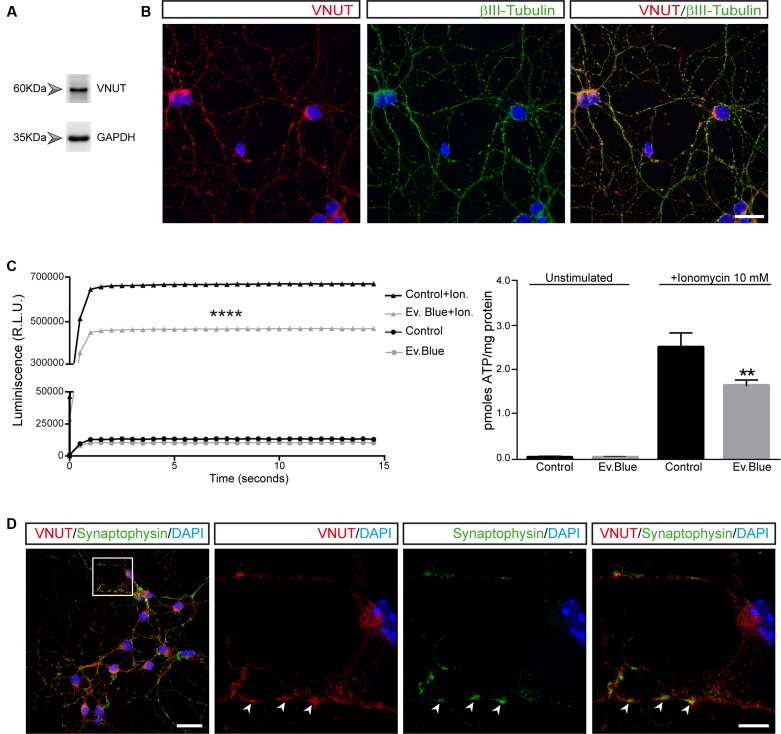
Vesicular nucleotide transporter (VNUT) is expressed by cultured cerebellar granule cells. Identification of VNUT in granule cells by western blotting **(A)** and immunofluorescence **(B)**. Cells were stained with antibodies against VNUT (red) and βIII-tubulin (green). Scale bar: 20 μm. **(C)** ATP release to the extracellular medium by granule cells. The graph shows the luminescence values of cells maintained in the presence or absence of Evans Blue (2 μM) and stimulated with ionomycin (10 μM) or left unstimulated. The right graph represents the pmoles of ATP released per mg of protein in the different experimental conditions. The values represent the mean ±SEM (*n* = 3: ^∗∗^*p*<0.01, ^∗∗∗∗^*p*<0.0001, unpaired Student’s *t*-test). **(D)** Representative confocal microscopy images of the immunostaining of granule cell neurons for VNUT (red) and synaptophysin (green). The nuclei are counterstained with DAPI (blue). Scale bar: 20 μm. The inset represents a 4x magnification of the cell indicated. The arrows indicate the most prominent VNUT and synaptophysin positive vesicles. Scale bar: 5 μm.

The expression of a variety of functional P2Y and P2X nucleotide receptors in primary cultures of cerebellar granule cells was already reported (Hervas et al., 2003, 2005; Leon et al., 2006; Sanchez-Nogueiro et al., 2009, 2014). In order to investigate the presence of native VNUT in granule neurons, western blot and immunofluorescence studies were performed. Unless specified otherwise, all experiments were carried out at day 7 *in vitro* coinciding with the peak of synaptogenesis in these cultured neurons (Mundy et al., 2008; Juranek et al., 2013). In western blots of total protein extracts from granule cells, a 60 kDa band was detected (**Figure 1A**) that is consistent with the molecular size of the VNUT protein (Sawada et al., 2008). The specificity of this band was confirmed using specific neutralizing peptides (data not shown). When immunocytofluorescence studies were performed on PFA-fixed cells using antibodies against VNUT and βIII-tubulin (**Figure 1B**), VNUT was widely expressed in granule cells displaying a characteristic punctuate staining of VNTs. The functionality of VNUT was also confirmed through the capacity of granule cells to release ATP in a calcium-dependent manner. Using the sensitive luciferin-luciferase assay to quantify ATP in the extracellular medium, the Ca^2+^-selective ionophore ionomycin produced a significant increase of extracellular ATP relative to the basal unstimulated state (**Figure 1C**). The kinetics of ATP release reflected a rapid mechanism, completed within seconds of stimulation and suggestive of calcium-dependent exocytosis. The participation of VNUT in vesicular ATP storage was confirmed using Evans Blue, a VNUT inhibitor (Sawada et al., 2008), which reduced the net release of ATP induced by ionomycin (**Figure 1C**). Remarkably, although Evans Blue was the most effective VNUT inhibitor available, previous studies in synaptic vesicles isolated from rat brain demonstrated that Evans Blue behaved only as a partial inhibitor of ATP release (Gualix et al., 1999b). Likewise, it is important to consider that inhibition of VNUT affects only the subsequent ATP transport and not the ATP stored prior to the treatment, which will be eventually released after ionomycin stimulation. The co-localization of VNUT and the vesicular protein synaptophysin further supported the exocytotic nature of ATP release (**Figure 1D**). Interestingly, co-localization of VNUT and synaptophysin immunostaining was not complete, indicating that VNUT may also be located in another type of storage vesicle or subcellular structure.

### Purinergic Receptor Activation Triggers the Release of ATP-Enriched Vesicles in Granule Cells

**FIGURE 2:**
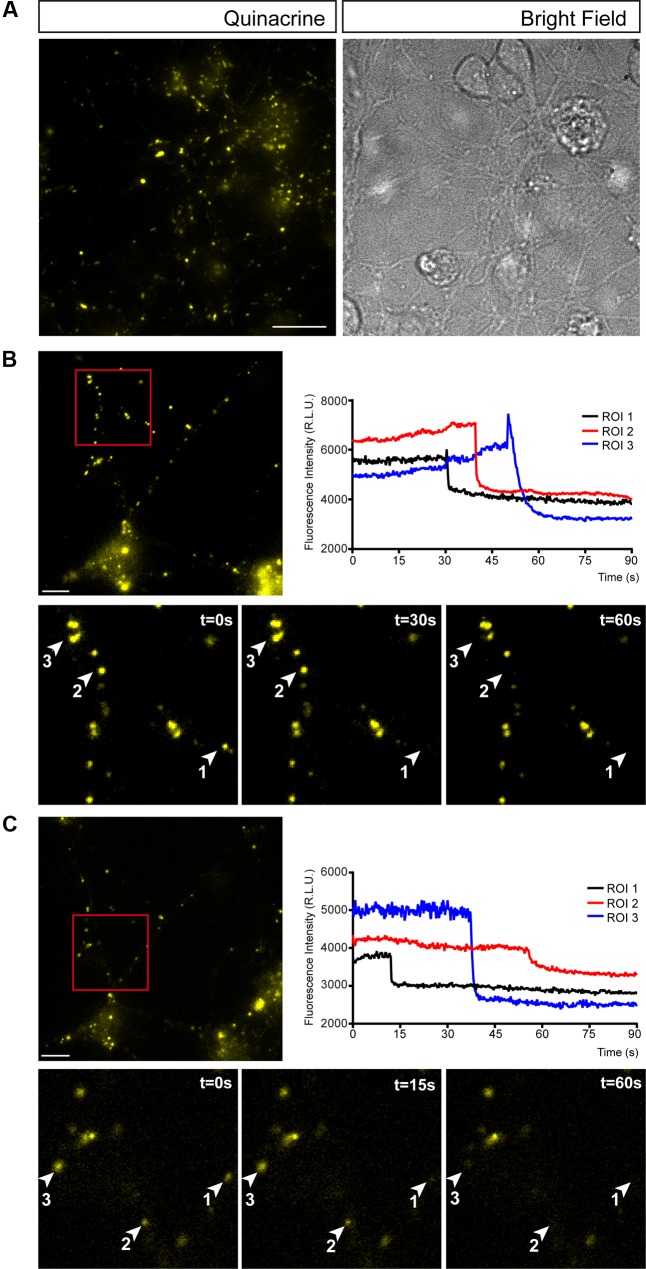
Cerebellar granule cells release ATP through by exocytosis. **(A)** Representative micrographs of granule cells stained with quinacrine (pseudo colored in yellow). Bright field image of the same area is showed on the right. Vesicular ATP release after stimulation with 1 mm ATP **(B)** or 30 mM KCl **(C)**. In each panel, the upper image corresponds to the micrograph representing quinacrine staining before stimulation. Scale bar: 5 μm. Insets represent sequential images of quinacrine-loaded vesicles showing the time course of the changes in fluorescence within a ROI during a 60 s stimulation with ATP **(B)** or KCl **(C)**. Graphical representation of the changes in fluorescence intensity corresponding to the selected ROIs during stimulation with ATP **(B)** or KCl **(C)**.

Many neuronal and non-neuronal cells release ATP in a controlled manner. Indeed, an autoregulated mechanism of ATP release has been reported where P2X7 receptor activation not only triggers ATP exocytosis but also, it profoundly modifies secretory vesicle dynamics in N2a neuroblastoma cells (Gutierrez-Martin et al., 2011). To assess whether similar autoregulation occurs in granule cells, we visualized exocytosis of single ATP-enriched vesicles from granule neurons after activation of purinergic receptors using fluorescence microscopy. Quinacrine is an acidophilic antimalarial drug that binds to intracellular ATP stored in vesicles and that was used as a fluorescent marker to detect the exocytosis of releasable ATP-enriched vesicles (Orriss et al., 2009; Liu et al., 2016). Granule cells contained abundant quinacrine-labeled vesicles and such punctate staining was not only evident throughout the soma but also, in cell prolongations (**Figure 2A**). Changes in fluorescence of single vesicles were analyzed under basal conditions and during ATP (1 mM) administration (**Figure 2B**). This ATP concentration was selected in order to trigger P2X7 receptors, which requires such a high concentration of ATP. As expected, ATP stimulation was followed by a reduction in the number of quinacrine fluorescence puncta (**Figure 2B**). Moreover, the abrupt time course of fluorescence loss in several regions of interest (ROIs) with quinacrine-stained vesicles reflected vesicular fusion with the plasma membrane and quinacrine release into the extracellular medium (**Figure 2B**). A parallel control assay was performed in which the cells were depolarized with 30 mM KCl, which produced a comparable release of quinacrine-stained vesicles (**Figure 2C**). These observations demonstrate that ATP-mediated activation of purinergic receptors can induce vesicular ATP release in cerebellar granule neurons.

### Subcellular Distribution of the VNUT in Cerebellar Granule Cells

**FIGURE 3:**
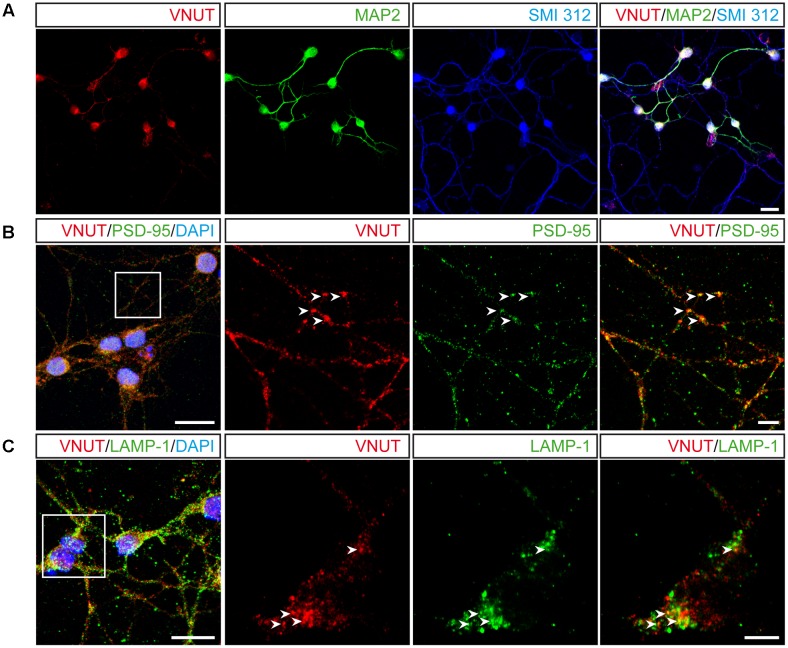
Subcellular localization of VNUT in cerebellar granule cells. **(A)** Representative confocal images showing the immunofluorescence for VNUT (red), MAP2 (green) and SMI 312 (blue). Scale bar: 20 μm. **(B)** Confocal images of double immunofluorescence for VNUT (red) and PSD95 (green). Scale bar: 15 μm. Insets represents higher magnifications to show the co-localization (yellow) of VNUT (red) and PSD95 (green). Arrowheads indicated co-localized immunostaining for the two proteins. Scale bar: 5 μm. **(C)** Representative confocal images of double immunofluorescence for VNUT (red) and LAMP-1 (green). Scale bar: 15 μm. Insets represents higher magnifications to show co-localization (yellow) of VNUT (red) and LAMP-1 (green). The arrowheads indicate co-localization of the immunostaining for the two proteins. Scale bar: 5 μm.

Given the morphological complexity of cerebellar granule cells in culture, we assessed the co-localization of VNUT with specific subcellular markers to define its distribution. When immunofluorescence was performed with antibodies against the somatodendritic marker microtubule-associated protein 2 (MAP2) and the pan-axonal neurofilament marker (SMI 312), VNUT co-localized with both these subcellular markers, indicating that this vesicular transporter exists both pre- and post-synaptically in granule neurons (**Figure 3A**). However, VNUT co-localized more intensely with MAP-2 than with SMI 312, suggesting that this vesicular transporter might be more strongly expressed post-synaptically. To confirm the presence of VNUT in dendrites, double immunofluorescence was performed with antibodies against VNUT and the postsynaptic density protein 95 (PSD-95) (**Figure 3B**). Both these proteins clearly co-localized, confirming that VNUT is located post-synaptically in granule neurons. The abundance of VNUT in the soma of granule cells suggested that this vesicular transporter is actively synthesized and packaged in earlier structures for further subcellular transportation, for instance into storage vesicles. Notably, VNUT expression has previously been reported in lysosomes (Shin et al., 2012; Jung et al., 2013; Oya et al., 2013; Cao et al., 2014) and interestingly, the clear co-localization in certain cytosolic areas of antibody staining for VNUT and the lysosomal marker LAMP-1 (**Figure 3C**), confirmed the presence of VNUT in lysosomes.

### Distribution and Time-Dependent Expression of VNUT and VGLUT1 in Primary Cultures of Cerebellar Granule Neurons

**FIGURE 4:**
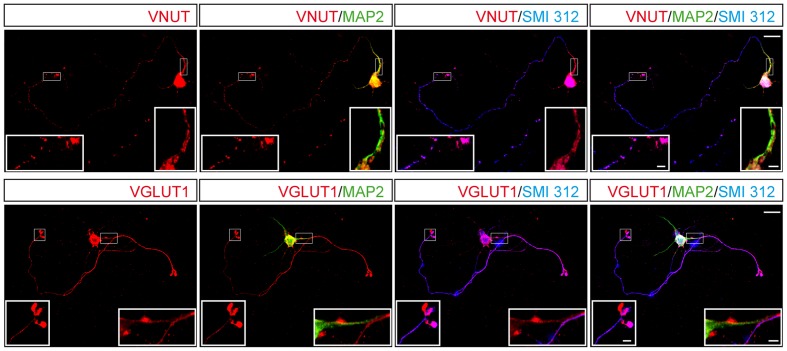
Distribution of VNUT or VGLUT1 in a single cerebellar granule cell. Representative confocal images showing the immunostaining for MAP2 (green), SMI 312 (blue) and either VNUT (red, upper panels) or VGLUT1 (red, lower panels). Scale bar: 15 μm. The insets represent higher magnifications of the somatodendritic or axonal compartments. Scale bar: 2.5 μm.

The glutamatergic phenotype of cerebellar granule cells reflects the storage and release of glutamate as a classic neurotransmitter, which requires the expression of VGLUTs, of which VGLUT1 is the most abundant isoform. Thus, we compared the subcellular distribution and time-dependent expression of this transporter and VNUT. Interestingly, the dense and abundant co-localization of VNUT and MAP2 was not accompanied by a similar co-localization between VGLUT1 and MAP2, which rarely coincided in dendritic regions (**Figure 4**). By contrast, while VNUT and SMI 312 co-localized sparsely in some prolongations, the co-localization of staining for SMI 312 and VGLUT1 was dense an almost complete (**Figure 4**).

**FIGURE 5:**
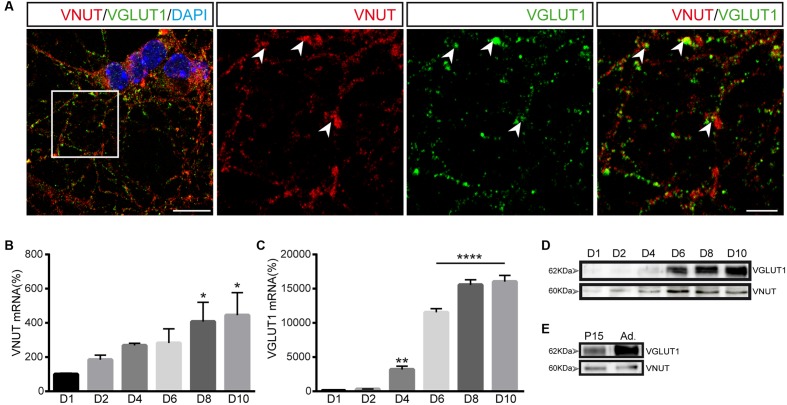
Expression of VNUT and VGLUT1 in cerebellar granule cells. **(A)** Representative confocal images showing double immunofluorescence for VNUT (red) and VGLUT1 (green). The nuclei were counterstained with DAPI (blue). Scale bar: 12 μm. Right panels represent a higher magnification to show the immunostaining for each vesicular transporter. The arrowheads indicate co-localization of the immunoreactivity for VNUT and VGLUT1. Immunofluorescence was performed on day 7 *in vitro*. Scale bar: 5 μm. VNUT **(B)** and VGLUT1 **(C)** mRNA levels analyzed by qPCR of cerebellar granule cells after different days of the culture. The values represent the mean ±SEM (*n* = 3: ^∗^*p*<0.05, ^∗∗^*p*<0.01, ^∗∗∗^*p*<0.001, ANOVA with Dunnet test with respect to day 1). **(D)** Western blots of the total protein (10 μg) in cell lysates after different times in culture. **(E)** Western blot of total protein extracts from cerebellum used as positive control of VNUT and VGLUT1 expression.

Double immunofluorescence was performed to determine whether VNUT and VGLUT1 coincide in the same vesicles and very few vesicles containing VGLUT1 were stained for VNUT, with most expressing only one of these vesicular transporters (**Figure 5A**). Thus, cerebellar granule cells appeared to contain segregated populations of secretory vesicles that store either ATP or glutamate. However, it should be remembered that these studies were performed *in vitro*, in primary cultures of granule neurons and, the expression of each transporter may vary during the maturation of the cells in culture. Thus, we analyzed the transcriptional and protein time-course of VNUT and VGLUT1 expression in these cultures from days 1 to 10 *in vitro.* While VNUT mRNA expression could be detected from the first day of culture and it gradually increased over the following days (**Figure 5B**), VGLUT1 expression was not evident until day 4 *in vitro*, although it increased strongly on the following days (**Figure 5C**). The detection of both these vesicular transporters in western blots correlated well with their mRNA expression (**Figure 5D**), consistent with the maturation of the culture and the establishment of synaptic contacts by granule cells *in vitro* and the development of a functional synaptic machinery. Cerebellar extracts from P15 or adult mice were used as positive control of both transporters (**Figure 5E**).

### Presence and Distribution of VNUT in the Mouse Cerebellar Cortex

**FIGURE 6:**
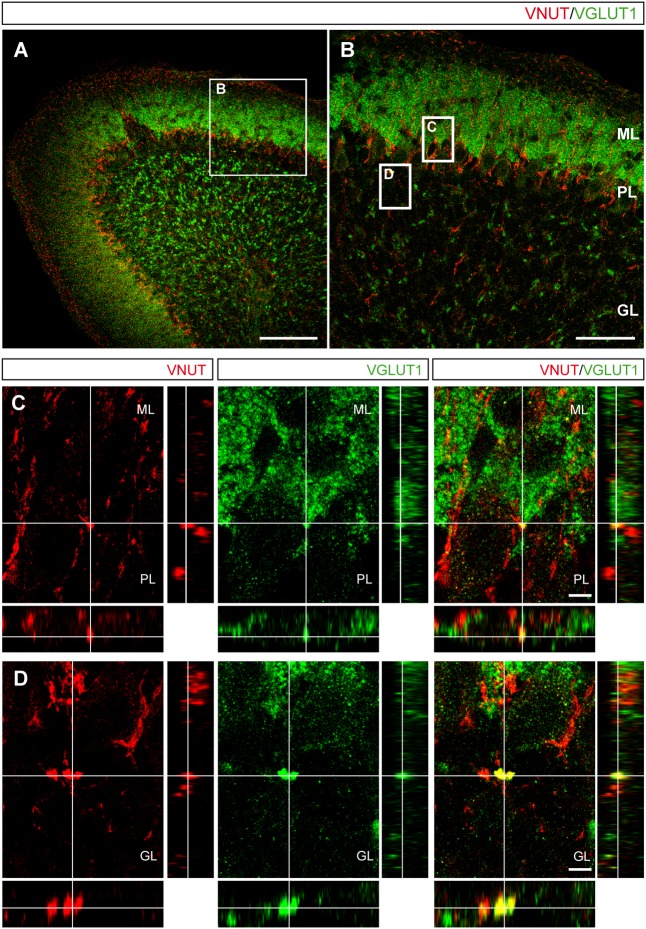
Localization of VNUT and VGLUT1 in the mouse cerebellar cortex. **(A)** Representative micrograph of a section of the P15 cerebellum immunostained for VNUT (red) and VGLUT1 (green). Scale bar: 100 μm. **(B)** Higher magnification of the selected region in **(A)** showing the three cerebellar layers. Scale bar: 50 μm. The insets are shown in the lower panels. **(C,D)** Orthogonal sections. Selected z-scanning series of images showing double labeling for VNUT and VGLUT1, representing the three planes of view for one point, as indicated by the crossed dashed lines. Scale bar: 10 μm. ML, Molecular Layer; PL, Purkinje Layer; GL, Granular Layer.

The aforementioned results in primary cultures of cerebellar granule cells suggested the existence of different vesicular pools preferentially containing either VNUT or VGLUT1, with a few vesicles exhibiting both transporters. To assess whether this distribution reflects the situation in the mouse cerebellum, this tissue was immunostained with antibodies against both vesicular transporters. In slices of the postnatal day 15 (P15) mouse cerebellum, both vesicular transporters were located in the molecular and the granular layers of the cerebellar cortex (**Figures 6A,B**). While VGLUT1 was found in the axons and soma of granule cells located in the molecular and granular layers, respectively (**Figure 6B**), VNUT was also observed in the Purkinje layer surrounding the soma of Purkinje neurons (**Figure 6B**). Although these vesicular transporters exhibited distinct distributions, confocal images with orthogonal views showed a few examples of co-localization between VNUT and VGLUT1 in both the molecular (ML) and granular (GL) layers (**Figures 6C,D**).

**FIGURE 7:**
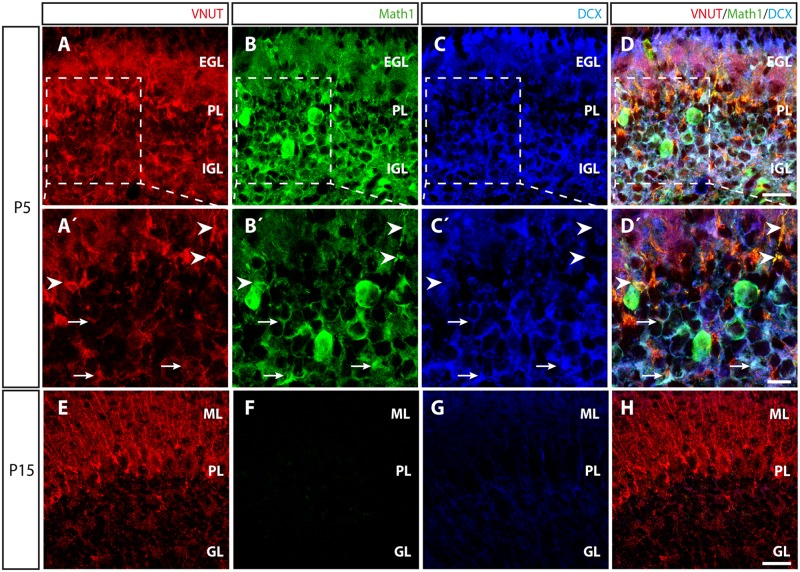
Distribution of VNUT during the postnatal development of the cerebellar cortex. **(A–D)** Representative confocal images of P5 cerebellar sections showing triple immunofluorescence for VNUT (red), Math1 (a marker of committed GCPs) (green) and DCX (a marker of immature neurons) (blue), and the overlaid image. Scale bar: 20 μm. **(A′–D′)** Higher magnification of the selected region at P5 in which the arrowheads indicate the co-localization of the immunoreactivity for VNUT and Math1. The arrows indicate the co-localization of VNUT, Math1 and DCX immunostaining. Scale bar: 10 μm. **(E–H)** Representative micrographs of P15 cerebellar sections showing triple immunofluorescence for VNUT (red), Math1 (green) and DCX, (blue) and the overlaid images. Scale bar: 20 μm. EGL, External Granular Layer; PL, Purkinje Layer; IGL, Internal Granular Layer; ML, Molecular Layer; GL, Granular Layer.

The presence of VNUT early in primary cultures of granule cells obtained from P5 mice (**Figures 1B**, **5B,D**) raised the question as to how such expression varied during cerebellar development. It is well known that the cortical layers of the cerebellum are not defined at P5 and that secondary proliferation niches containing granule cell precursors (GCPs) can be found (Altman, 1972; Espinosa and Luo, 2008). Moreover, at this time immature granule cells are migrating from the external granular layer (EGL) to the internal granular layer (IGL), where they will reach their final location (Altman and Bayer, 1997). At P5, VNUT staining in cerebellar slices was stronger and more widely distributed (**Figures 7A,A′**) than at P15 stage (**Figure 7E**). To characterize the specific populations expressing VNUT during development, we used an antibody against Math1 to identify GCPs, a transcription factor of the bHLH (basic helix-loop-helix) class that is essential for the proper development of cerebellar granule layer (Ben-Arie et al., 1997), and an antibody against the microtubule-associated protein doublecortin (DCX) to recognize neuroblasts and immature neurons (**Figure 7**). At P5, VNUT and Math1 co-localized in the cerebellum (**Figures 7D,D′**), confirming that GCPs express VNUT during their postnatal development. Moreover, in certain regions of the IGL, VNUT, Math1 and DCX co-localization was also observed (**Figures 7D,D′**). Hence, the onset of VNUT expression appears to commence early in cerebellar development, and it is expressed by neuronal progenitors like GCPs and immature neurons that have not fully differentiated. Alternatively, Math1 and DCX expression was weaker at P15 due to the development of the cerebellum (**Figures 7E–H**), although VNUT persisted at this stage and it was found in several cerebellar layers, yet mainly in the ML. These results correlate with our observations *in vitro*, whereby VNUT is expressed from the first day of cerebellar granule cell culture, when neurons are still immature, persisting once the neurons have matured and established functional synapses.

## Discussion

The present study confirms that the VNUT, is functionally expressed in primary cultures of cerebellar granule neurons, its expression varying as the cell cultures mature *in vitro* and it is distributed in presynaptic and postsynaptic vesicles in these neurons. Moreover, we demonstrate that VNUT is expressed very early in the cerebellar granule neuronal lineage, both *in vitro* and *in vivo*. Together, these results imply that VNUT potentially contributes to the postnatal development of granule cells in the cerebellar cortex, a role that will be challenging to define.

Extracellular nucleotides play an essential role in cellular signaling, acting through purinergic receptors, and in neural tissues, extracellular ATP is involved in physiological processes like synaptic transmission, proliferation, differentiation or axonal elongation (Diaz-Hernandez et al., 2008; Gomez-Villafuertes et al., 2009; Diez-Zaera et al., 2011). In physio-pathological conditions including neuropathic pain, psychiatric disorders or epileptic seizures, enhanced P2 receptor expression has been reported, such as P2X4 or P2X7 (Tsuda et al., 2003; Beggs et al., 2012; Henshall et al., 2013; Sperlagh and Illes, 2014). Thus, the levels of extracellular nucleotides must be tightly controlled in order to only activate specific purinergic receptors when necessary. This tight regulation mainly focuses on controlling the release and the removal of nucleotides from the extracellular space. In terms of extracellular nucleotide removal, these compounds can be rapidly hydrolyzed to the last product adenosine, subsequently transported and incorporated to the cellular nucleotide pool (Rotllan and Miras Portugal, 1985; Miras-Portugal et al., 1986; Sen et al., 1998; Zimmermann et al., 2012; Gomez-Villafuertes et al., 2014; Pastor-Anglada and Perez-Torras, 2015).

By contrast, the exocytotic release of ATP and other nucleotides depends on VNUT activity, which links the intra- and extracellular steps of ATP signaling. Although its existence and kinetic parameters were described long ago (Bankston and Guidotti, 1996; Gualix et al., 1996, 1999a), not until recently was VNUT cloned (Sawada et al., 2008). VNUT is heterogeneously expressed in distinct cell subtypes and structures in the murine brain, being particularly abundant in cerebellar cortex (Larsson et al., 2012). However, little is known about its specific sub-cellular distribution and function within the complex cyto-architecture of the cerebellum.

Among the different neuronal subtypes that make up the cerebellum, granule cells are the most abundant. Purinergic signaling seems to play an important role in these cells, since they not only strongly express different purinergic receptors but also, VNUT (Larsson et al., 2012). Primary cultures of granule cells represent a suitable model to understand basic neurobiological processes. For instance, depolarization of these glutamatergic neurons through the activation of P2X3 or P2X7 receptors can induce glutamate release (Leon et al., 2008). However, as mentioned previously, there is nothing known about the role of VNUT in this cell model, although the data presented here clarifies some relevant issues. First, VNUT appears to co-localize with the vesicular marker synaptophysin in these cells and in addition, granule cells appear to release ATP by exocytosis. As inhibiting VNUT reduces the net release of ATP, VNUT is clearly involved in the vesicular storage of ATP as indicated elsewhere (Gualix et al., 1999a,b; Sawada et al., 2008).

We previously showed that activation of P2X7 receptors by ATP evokes the release of this nucleotide in neuroblastoma N2a cells (Gutierrez-Martin et al., 2011), and here we demonstrate a similar phenomenon occurs in cerebellar granule cells. Quinacrine loading highlighted the large number of acidic fluorescent vesicles that store ATP in cerebellar granule cells, and ATP stimulation induced an abrupt loss of fluorescence due to exocytosis. Hence, ATP appears to induce its own release in granule cells, an effect that could be mediated by P2X7 receptors since they require high ATP concentrations to be activated (Gutierrez-Martin et al., 2011). Depolarization induced by KCl also confirms the existence of a vesicular mechanism for ATP release, although it does not produce complete loss of quinacrine-loaded vesicles. This may indicate that not all the quinacrine-loaded vesicles belong to the ready releasable pool close to the plasma membrane or that these vesicles are not secretory ones. This latter possibility was confirmed by immunofluorescence assays with the lysosomal marker LAMP-1, whereby quinacrine-loaded vesicles that do not respond to the increase in intracellular calcium could be “conventional” lysosomes that accumulate high concentrations of ATP (Li et al., 2008). Moreover, VNUT does not fully co-localize with synaptophysin, indicating that the vesicular transporter is present in non-synaptic vesicles. Indeed, VNUT has been identified in lysosomes of different cellular lineages (Shin et al., 2012; Oya et al., 2013; Cao et al., 2014), including sensory neurons (Jung et al., 2013). Lysosomes are best known for their role in protein degradation during autophagy and phagocytosis, yet they may also be involved in regulated exocytosis, acting as secretory vesicles (Blott and Griffiths, 2002; Li et al., 2008). Moreover, lysosomes can store large amounts of ATP and release it through exocytosis (Zhang et al., 2007; Pryazhnikov and Khiroug, 2008; Dou et al., 2012). In terms of cerebellar granule cells, the co-localization between VNUT and LAMP-1 confirmed the presence of this vesicular transporter in lysosomes, suggesting that these neurons can store ATP in lysosomes, and even release it from these vesicles by exocytosis.

Several studies of vesicular ATP release at central synapses suggest that ATP comes from a different pool of vesicles that is not synchronized either with GABA or glutamate release (for review see Pankratov et al., 2006). To assess whether cerebellar granule cells that release ATP and glutamate, and that express both VNUT and VGLUT1, also have a specific pool of ATP-containing vesicles, the distribution of both vesicular transporters was studied. When compared with the somatodendritic marker MAP2 and the neurofilament axonal marker SMI 312, VNUT was clearly present in both pre- and postsynaptic areas. By contrast, VGLUT1 was mainly seen to co-localize with the axonal marker and it was barely present in the somatodendritic regions. On the other hand, co-localization of VNUT and PSD-95 confirmed the transporter’s presence in postsynaptic domains, suggesting that exocytosis of ATP could be bi-directional at synaptic contacts. Accordingly, the ATP released in the synaptic cleft can activate both pre- and postsynaptic purinergic receptors. At the presynaptic level, P2X receptor activation induces or enhances neurotransmitter release (Gu and MacDermott, 1997; Khakh and Henderson, 1998; Nakatsuka and Gu, 2001; Khakh et al., 2003; Rodrigues et al., 2005; Sperlagh et al., 2007; Khakh, 2009), whereas P2Y receptor activation mainly dampens neurotransmitter release (Rodrigues et al., 2005). Additionally, activation of postsynaptic P2X receptors may induce AMPA receptor internalization at the dendrites (Pougnet et al., 2014), modulating synaptic transmission. Presynaptic modulation by ATP could originate from different sources, the terminal itself and neighboring nerve terminals or astrocytes. Moreover, the postsynaptic location of VNUT in granule cells suggests an additional source of ATP through its release from dendrites. VNUT was previously found in postsynaptic dendrites of dentate gyrus and hippocampal CA1 neurons (Larsson et al., 2012). The possibility that dendritic spines might be able to release ATP indicates that they might modulate presynaptic release of neurotransmitters through a retrograde mechanism. Exocytotic release of ATP and glutamate from granule cells can occur simultaneously, although they may be stored in different vesicle pools, as reflected by the segregated distribution of VNUT and VGLUT1. Indeed, co-localization between these vesicular transporters was rare after 7 days *in vitro*, although the expression of VNUT and VGLUT1 increased as the granule cell cultures mature, consistent with the establishment of synaptic contacts that require the corresponding synaptic machinery. Nevertheless, VNUT expression by these cells was detected very early, from their first day in culture.

As the granule cells culture were obtained at an early postnatal developmental stage, it is possible that the expression of VNUT and VGLUT1is distinct in the native tissue. Thus, the distribution of both vesicular transporters was analyzed in sagittal sections of the cerebral cortex at P15 stage, when VGLUT expression increases significantly (Boulland et al., 2004). The two vesicular transporters exhibited a clear non-overlapping distribution, with scarce co-localization, which was consistent with the results obtained in granule cell cultures. In fact, while the two transporters are located in both the molecular and granule layer of the cerebellar cortex, VNUT-staining was stronger in the molecular stratum. On the other hand, VNUT was also located around the soma of Purkinje cells, suggesting an important role of purinergic signaling in controlling GABAergic transmission (Casel et al., 2005). Indeed, it has already been demonstrated that P2X receptors can elicit Ca^2+^ responses in primary cultures of Purkinje cells (Mateo et al., 1998).

The evolution of VNUT expression in the mouse cerebellum was assessed from the immature postnatal P5–P15 stage. As the cerebellar layers are not well defined at P5 and granule cells are still migrating from the external to the internal granular layer, this appears to be a good model to correlate VNUT expression with that of the essential specific factors, Math1 and DCX. Math1 is a master gene that specifies the cerebellar granule neuron lineage and it is expressed in the committed GCPs (Ben-Arie et al., 1997; Srivastava et al., 2013), whereas DCX is a microtubule-associated protein that identifies neuronal precursors and immature neurons (Brown et al., 2003). In accordance with its early expression *in vitro*, VNUT was expressed abundantly at P5, and it co-localized with both Math1 and DCX, suggesting that it is expressed by GCPs and the immature neurons migrating from the external to the internal granular layer of the cerebellum. During the subsequent development of the cerebellum, committed granule cells become mature granule neurons between P5 and P15, as confirmed by the absence of Math1 and the dramatic reduction in DCX. Nevertheless, VNUT remains prominently expressed in the molecular and granule layer, highlighting the relevance of VNUT during the commitment and differentiation of cerebellar granule neurons.

There is now increasing evidence implicating VNUT in both physiological and pathological processes in neural tissues. For instance, VNUT helps regulate neuronal differentiation of neuroblastoma N2a cells (Menendez-Mendez et al., 2015) Moreover, during the development of glaucoma, there is a direct correlation between enhanced VNUT expression in the intern plexiform layers of the retina and an increase in extracellular ATP (Perez de Lara et al., 2015). More recently it was proposed that VNUT-dependent ATP release from spinal cord dorsal root neurons might originate neuropathic pain in mice (Masuda et al., 2016). Furthermore, clodronate was identified as a novel inhibitor of VNUT for the treatment of neuropathic and inflammatory pain, opening up new therapeutic possibilities for other pathologies (Kato et al., 2013, 2017).

In summary, we have characterized the subcellular localization and activity of VNUT during the maturation of cerebellar granule neurons *in vitro.* The correlation between these findings and the situation in the cerebellum *in vivo* raises exciting new questions that should be addressed in the near future.

## Author Contributions

AM-M designed, performed the majority of experiments and wrote the manuscript. JD-H designed and supervised the experiments. FO designed, performed the experiments and wrote the manuscript. JG designed and supervised the experiments. RG-V and MM-P was designed, supervised the experiments and wrote the manuscript.

## Conflict of Interest Statement

The authors declare that the research was conducted in the absence of any commercial or financial relationships that could be construed as a potential conflict of interest. The reviewer MP-A and handling editor declared their shared affiliation.
